# Signal-transducing adaptor protein 1 (STAP1) in microglia promotes the malignant progression of glioma

**DOI:** 10.1007/s11060-023-04390-8

**Published:** 2023-07-18

**Authors:** Xinyu Yang, Chunxia Ji, Ying Qi, Jianhan Huang, Lang Hu, Yuan Zhou, Liping Zou, Yi Xia, Feng Tan, Yu Yao, Di Chen

**Affiliations:** 1 Department of Neurosurgery, Huashan Hospital, Shanghai Medical College, Fudan University, Shanghai, China; 2 National Center for Neurological Disorders, Shanghai, China; 3 Shanghai Key Laboratory of Brain Function and Restoration and Neural Regeneration, Shanghai, China; 4 Immunology Laboratory, Neurosurgical Institute of Fudan University, Shanghai, China; 5 Shanghai Clinical Medical Center of Neurosurgery, Shanghai, China; 6 Department of Pathology, Huashan Hospital, Fudan University, Shanghai, China; 7 School of Basic Medical Sciences, Wenzhou Medical University, Wenzhou, Zhejiang China

**Keywords:** STAP1, Glioma, Microglia, Polarisation

## Abstract

**Background:**

Glioma is the most malignant primary brain tumor with a poor survival time. The tumour microenvironment, especially glioma-associated microglia/macrophages (GAMs), plays an important role in the pathogenesis of glioma. Currently, microglia (CD11b^+^/CD45^Low^) and macrophages (CD11b^+^/CD45^High^) are distinguished as distinct cell types due to their different origins. Moreover, signal-transducing adaptor protein 1 (STAP1) plays a role in tumourigenesis and immune responses. However, to date, no studies have been reported on STAP1 in GAMs.

**Methods:**

The Cancer Genome Atlas and Chinese Glioma Genome Atlas databases were used to investigate the association between STAP1 mRNA levels and clinical parameters (grades, mutations in isocitrate dehydrogenase, and overall survival). RNA-sequencing, qRT-PCR, Western blotting, immunohistochemistry and immunofluorescence analyses were performed to detect the expression level of STAP1 and related proteins. BV-2 cells were used to construct a STAP1-overexpressing cell line. Phagocytosis of BV-2 cells was assessed by flow cytometry and fluorescence microscopy. C57BL/6 mice were used to establish orthotopic and subcutaneous glioma mouse models. Glioma growth was monitored by bioluminescence imaging.

**Results:**

STAP1 expression in glioma-associated microglia is positively correlated with the degree of malignancy and poor prognosis of glioma. Moreover, STAP1 may promote M2-like polarisation by increasing ARG1 expression and inhibiting microglial phagocytosis of microglia. Increased ARG1 may be associated with the IL-6/STAT3 pathway. Impaired phagocytosis may be associated with decreased cofilin and filopodia.

**Conclusion:**

STAP1 is positively associated with the degree of glioma malignancy and may represent a potential novel therapeutic target for glioma.

**Supplementary Information:**

The online version contains supplementary material available at 10.1007/s11060-023-04390-8.

## Introduction

Glioma, the most common primary malignant brain tumour, is characterized by high mortality [1]. Currently, the main therapies used to treat glioma include surgical resection, chemotherapy, radiotherapy, and tumor-treating fields. However, the therapeutic outcomes and survival rates remain unsatisfactory [2]. Tumour heterogeneity and the tumour microenvironment (TME) play a critical role in the development and progression of glioma [3]. Tumour heterogeneity is the main reason for the failure of targeted therapies, and the TME reprograms tumour cells [4]. Therefore, many researchers have focused on the TME, mainly including immune cells, cytokines, and the extracellular matrix [5]. Tumour-associated microglia and macrophages (TAMs), the most abundant immune cells in many cancers, are critical factors in tumour-associated immune responses [6]. Glioma-associated microglia/macrophages(GAMs), which comprise up to 30% of the tumour mass, facilitate immunosuppression and pathological angiogenesis in GBM [7]. Moreover, resident microglia, rather than peripheral macrophages, represent a major proportion of the early stage of glioma, whereas macrophages infiltrate in the late stage [8, 9]. In different microenvironments, microglia would be reprogramed towards different phenotypes, which can be simply divided into an M1-like phenotype (activation state) and an M2-like phenotype (alternative activation state) [10]. The M1-like phenotype promotes an inflammatory response, whereas the M2-like phenotype inhibits the inflammatory response and promotes tumour progression [11]. Blocking certain targets and restoring normal functions of the microglia, to some extent, may reduce glioma expansion and prolong overall survival (OS) [12]. Therefore, it is important to investigate the effects of GAMs and their role as therapeutic targets to develop new targeted drugs with better clinical efficacy.

Owing to the similar biological functions of microglia and immature research techniques, only a few studies focused on differentiating macrophages and microglia. Macrophages originate from the bone marrow and express high levels of CD45, whereas microglia originate from the yolk sac and express low levels of CD45 [13]. A study based on single-cell RNA sequencing has revealed differences between microglia and macrophages in gene expression [14]. Multi-omics studies have shown that microglia are always present in normal brain tissues and glioma tissues, whereas macrophages infiltrate gliomas only when the blood-brain barrier is disrupted [15–17]. Therefore, microglia may play a more important role in the occurrence and progression of gliomas.

The signal-transducing adaptor protein (STAP) family, which includes STAP1 and STAP2, regulates various intracellular signalling pathways involved in tumourigenesis and immune responses. Based on the Brain Cancer Immunology Atlas (BCIA) at Huashan Hospital, Fudan University, we found that STAP1 is differentially expressed in different grades of glioma. STAP1 was first named B cell antigen receptor downstream signalling 1 based on a yeast two-hybrid screening [18]. STAP1 and STAP2 inhibit apoptosis of leukaemic stem cells in chronic myeloid leukaemia by binding to BCR-ABL fusion oncoprotein and signal transducer and activator of transcription 5a (STAT5a) [19, 20]. Moreover, STAP2 promotes breast cancer cell growth by interacting with STAT3, which is closely associated with tumours [21]. However, compared to STAP2, there have been few studies on STAP1, and to date, no studies on STAP1 in GAMs and glioma have been reported.

The aim of the present study was to investigate the relationship between STAP1 expression levels and the degree of glioma malignancy. We also investigated the molecular mechanisms that promote glioma progression.

## Materials and methods

### Patient samples

All samples were obtained continuously from the Department of Neurosurgery, Huashan Hospital, Fudan University. The use of these samples was approved by the Medical Ethical Committee of the Huashan Hospital. GAMs used for transcriptome sequencing were obtained between 2016 and 2019. Glioma samples used for immunohistochemistry and their GAMs used for Western blotting and real-time quantitative reverse transcription PCR (qRT-PCR) were continuously obtained between 2020 and 2021 (Ethics Number: KY2020-009) (Table. S1). The tissue microarray (TMA) section was obtained as described in our previous study [22].

### GAMs isolation

As shown in the flowchart (Fig. S1a), glioma tissues were minced with scissors, digested with acctuase (SCR005, Sigma), and filtered into a single-cell suspension in a 40-µm cell strainer (352,340, Falcon). After removal of red blood cells using red blood cell lysis buffer (B541001, Sangon Biotech), cell suspension was diluted in magnetic affinity cell sorting (MACS) buffer (130-091-221, Miltenyi Biotec) and incubated with CD11b microbeads (130-093-634, Miltenyi Biotec) for 15 min at 4 ℃. The cell suspension was applied to the MS column (130-042-201, Miltenyi Biotec). CD11b-positive cells were then labelled by a washing column. After pressing the plunger firmly into the column, positive cells were eluted and collected [23]. Flow cytometry was used to verify the cell purity (Fig. S1 b, c).

### Cell culture and transfection

GL261 glioma cells transfected with firefly luciferase expression vector, GL261-luc cells, were kindly donated by Sichuan University. BV-2 microglia cells were donated by Sun Yat-sen University. To stably express STAP1, BV-2 cells were transfected with lentivirus with pHBLV-EF1-STAP1-Flag-CMV-puro vector (Hanheng Biotechnology), designated BV-2-STAP1 and cells transfected with empty vectors were designated BV-2-CON (Fig. S5a, b). After 48 h of transfection, BV-2-STAP1 and BV-2-CON cells were treated with puromycin (5 µg/mL; Ant-pr, Invivogen) for 5 d and resistant cells are selected. Cells were grown in Dulbecco’s modified Eagle’s medium (SH30243.01, Hyclone) supplemented with 10% fetal calf serum (10,099,141 C, Gibco), penicillin, and streptomycin (15140-122, Gibco). Cells were cultured in a 37 ℃ incubator with a 5% CO_2_ atmosphere.

### Orthotopic and subcutaneous glioma mice models

Adult female C57BL/6 mice (6 weeks old, 18–22 g) were purchased from the Jiesijie Experimental Animal Company (Shanghai, China). For orthotopic glioma models, mice were anaesthetised with 1.25% avertin (150µL/10 g; M2920, Nanjing AIBI Bio-Technology) and placed on a stereotaxic apparatus (Ruiwode Shenzhen). To develop the orthotopic glioma model, 5 × 10^4^ GL261-luc cells mixed with 1.25 × 10^4^ BV-2-CON cells or BV-2-STAP1 cells were injected into the right striatum. For subcutaneous glioma models, 1 × 10^6^ GL261-luc cells mixed with 2.5 × 10^5^ BV-2-CON cells or BV-2-STAP1 cells were injected into the right dorsum subcutaneously of the mice. Animal experiments were approved by the Department of Laboratory Animal Science of Fudan University (Ethics Number: 201906007s).

### Public data access

RNA-sequencing data, clinical information and molecular data were obtained from The Cancer Genome Atlas (TCGA) (https://tcga-data.nci.nih.gov/tcga/) and Chinese Glioma Genome Atlas (CGGA) (http://www.cgga.org.cn). The inclusion criteria and exclusion criteria are shown in Fig. S3.

### Statistical analysis

Data were obtained from at least three independent experiments. Data with a normal distribution were analyzed by T-test. Otherwise, Mann-Whitney U-test was used. Survival curves are plotted by the Kaplan-Meier method and compared by log-rank tests. The significance levels are as follows: NS (no significance); * (*P* < 0.05); ** (*P* < 0.01); *** (*P* < 0 0.001); **** (*P* < 0 0.0001).

.

## Results

### STAP1 is a prognostic factor that is associated with the degree of malignancy of glioma

Based on isolated GAMs (Fig. S1), we performed transcriptome sequencing of GAMs and found that *STAP1* mRNA levels were higher in GAMs of grade 4 glioma than in grade 2–3 glioma (Fig. S2). Furthermore, we used the TCGA and CGGA public datasets and then divided patients with glioma into a low-STAP1 group and a high-STAP1 group according to the median mRNA levels of *STAP1*. The inclusion criteria and exclusion criteria were listed in Fig. S3. Kaplan-Meier survival analysis showed that patients in low-STAP1 group had a higher overall survival (OS) than those in the high-STAP1 group (Fig. S4a). Similarly, low expression level of STAP1 prolonged the OS of patients with grade 2–3 glioma (Fig. S4b). Although difference was not statistically significant, the similar trend could also be seen in patients with grade 4 glioma (Fig. S4c). These fundings indicate that STAP1 may be associated with the degree and poor prognosis of malignancy of glioma.

**Fig. 1:**
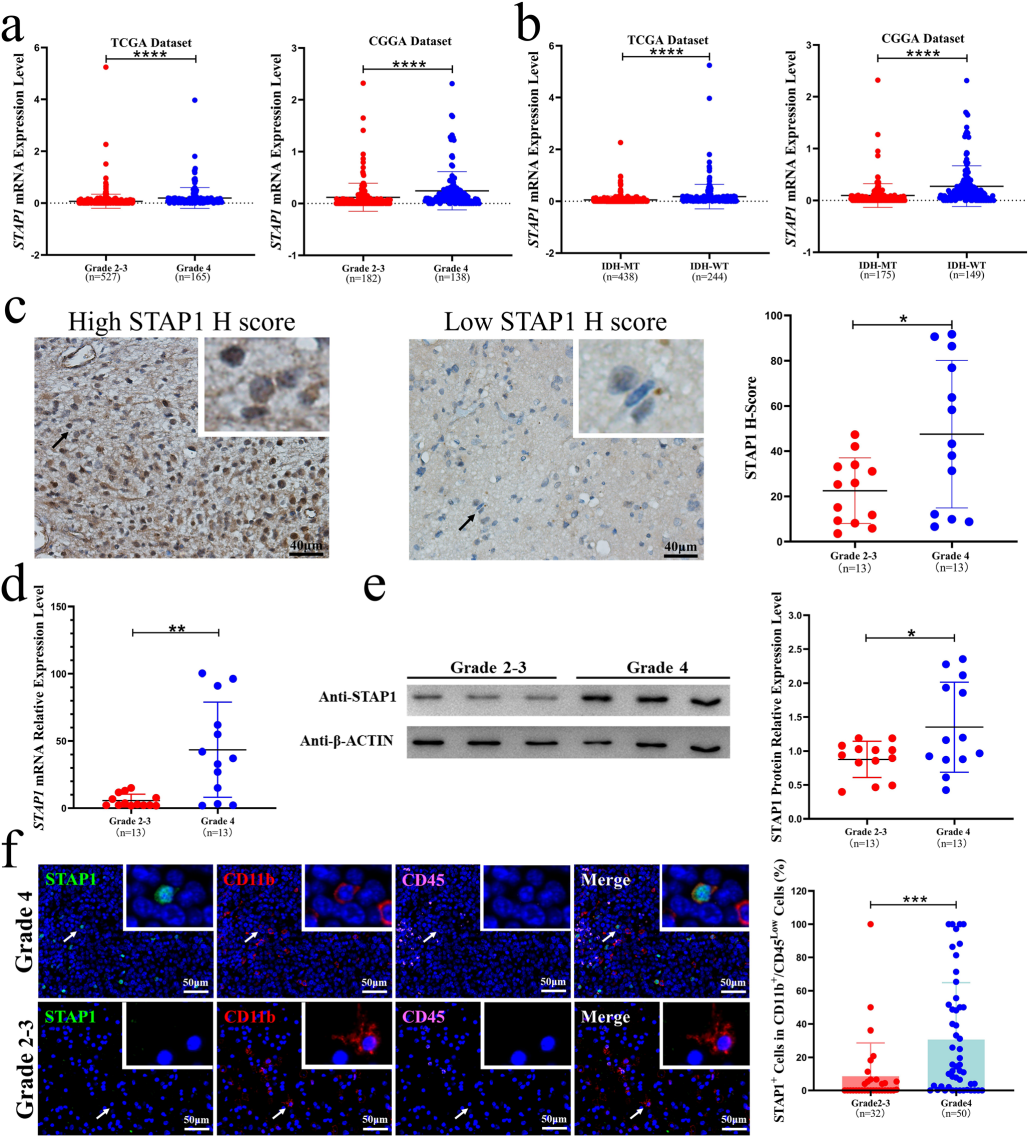
STAP1 expression level is associated with the degree of malignancy and prognosis of glioma. **a** mRNA levels of *STAP1* in patients with different grades of glioma in TCGA datasets and CGGA datasets. U-test. **b** mRNA levels of *STAP1* were higher in IDH wild type gliomas than in IDH mutant type gliomas in TCGA datasets and CGGA datasets. U-test. **c** Histochemistry score of STAP1 was higher in grade 4 glioma than grade 2–3 glioma. Scale bars, 20 μm. T-test. **d** qRT-PCR analysis found the relative mRNA levels of STAP1 in grade 4 GAMs was higher than those in grade 2–3 GAMs. T-test. **e** Western blot analysis found the relative protein levels of STAP1 were higher in grade 4 GAMs than in grade 2–3 GAMs. T-test. **f** Immunofluorescence analysis found the protein expression level of STAP1 in grade 4 glioma-associated microglia was higher than grade 2–3 glioma associated microglia. Scale bars, 50 μm. U-test. *P < 0.05, **P < 0.01, ***P < 0.001, ****P < 0.0001

To validate this result, we assessed the transcript levels of *STAP1* in glioma samples from the TCGA and CGGA datasets. The inclusion and exclusion criteria were listed in Fig. S3. Compared to grade 2–3 gliomas, the mRNA levels of *STAP1* were higher in grade 4 gliomas (Fig. 1a). Moreover, isocitrate dehydrogenase (IDH) wild type glioma, a subtype associated with poor outcomes, showed higher expression levels of STAP1 than IDH mutant glioma (Fig. 1b). We then collected 26 glioma tissue samples for immunohistochemistry (Table S1) and the protein level of STAP1 was significantly higher in patients with grade 4 glioma than those with grade 2–3 glioma (Fig. 1c). These data indicate that STAP1 may be associated with the degree of malignancy of glioma.

Given the important role of GAMs in the TME, we collected 30 GAMs for qRT-PCR and western blotting to determine the distribution of STAP1 in GAMs and microglia (Table S1). qRT-PCR showed that *STAP1* mRNA levels were higher in grade 4 GAMs than in grade 2–3 GAMs (Fig. 1d). At the protein level, western blot analysis showed that STAP1 was also higher in grade 4 GAMs than in grade 2–3 GAMs (Fig. 1e). Recent studies on GAMs have distinguished between microglia and macrophages separately [24]. Therefore, we investigated STAP1 protein expression profiles in microglia. Immunofluorescence staining showed a higher expression level of STAP1 in the microglia of grade 4 gliomas than those of grade 2–3 gliomas (Fig. 1f). These data suggest that STAP1 expression in the microglia may play a key role in promoting the malignant progression of glioma.

### STAP1 promotes microglia M2-like polariation by increasing ARG1 expression

**Fig. 2:**
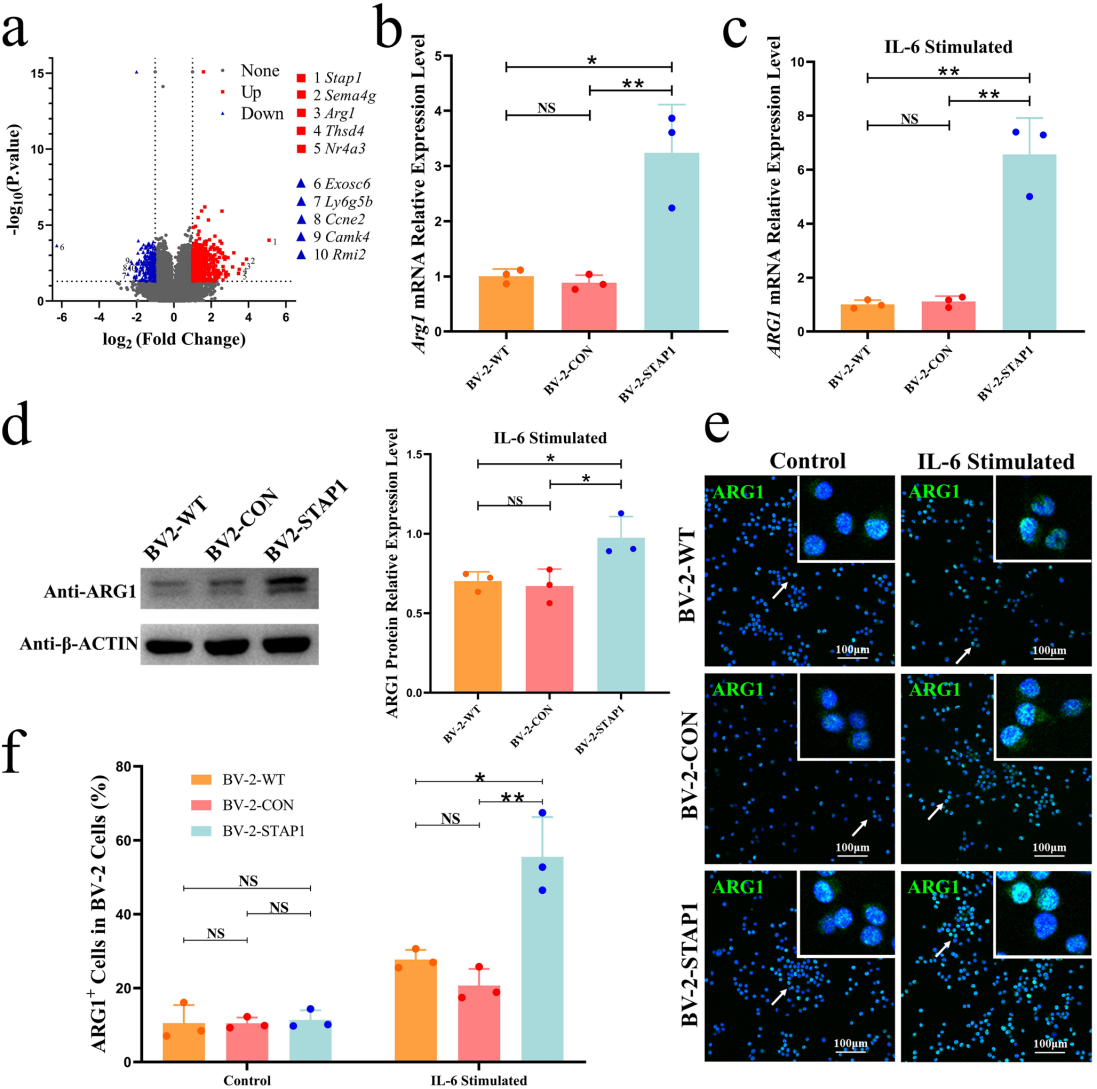
STAP1 Overexpression Promotes Microglia M2-like Polarisation by Increasing ARG1 Expression. **a** Volcano plot showing the changes of gene expression in BV-2-STAP1 cells compared to BV-2-CON cells. Transcriptomic sequencing revealed that the relative mRNA level of *Arg1* was higher in BV-2-STAP1 than BV-2-CON cells. T-test. **b** qRT-PCR analysis revealed that the relative mRNA level of *Arg1* was higher in BV-2-STAP1 cells. T-test. **c** After IL-6 (25ng/mL) stimulation, mRNA level of *Arg1* was obviously increased in BV-2-STAP1 cells. T-test. **d-f** Western blot and immunofluorescence analyses showed that, after IL-6 stimulation, STAP1 protein level was obviously increased. Scale bars, 100 μm. T-test. *P < 0.05, **P < 0.01

To explore the biological functions of STAP1 in microglia, we established BV-2-STAP1 cells to stably overexpress STAP1 and BV-2-CON (cells transfected with empty vectors) as a control group (Fig. S5). The volcano plot of the transcriptome sequencing showed that the expression of the key marker of M2-like polarisation, *Arg1*, increased by 3.7-fold in BV-2-STAP1 cells compared to that in BV-2-CON cells (Fig. 2a). Similarly, qRT-PCR showed that *Arg1* expression was significantly increased in BV-2-STAP1 cells compared to that in BV2-CON cells (Fig. 2b). However, Western blot analysis showed that ARG1 was slightly up-regulated at the protein level, with no statistical significance (Fig. S6).

A previous study has shown that, interleukin-6 (IL-6), a tumour-promoting cytokine in the TME, can induce microglia M2-like polarisation by increasing ARG1 expression [25]. Therefore, we determined ARG1 expression after stimulating the cells with IL-6 and found that, after exposure to IL-6, both the transcriptional and translational levels of *Arg1* were significantly increased in BV-2-STAP1 compared to those in BV-2-WT and BV-2-CON (Fig. 2c, d). We further confirmed these results using immunofluorescence staining (Fig. 2e, f). These results suggest that, in response to IL-6 stimulation, STAP1 promotes ARG1 expression, which indicates an M2-like phenotype.

### STAP1 promotes ARG1 expression via the IL-6/STAT3 pathway

**Fig. 3:**
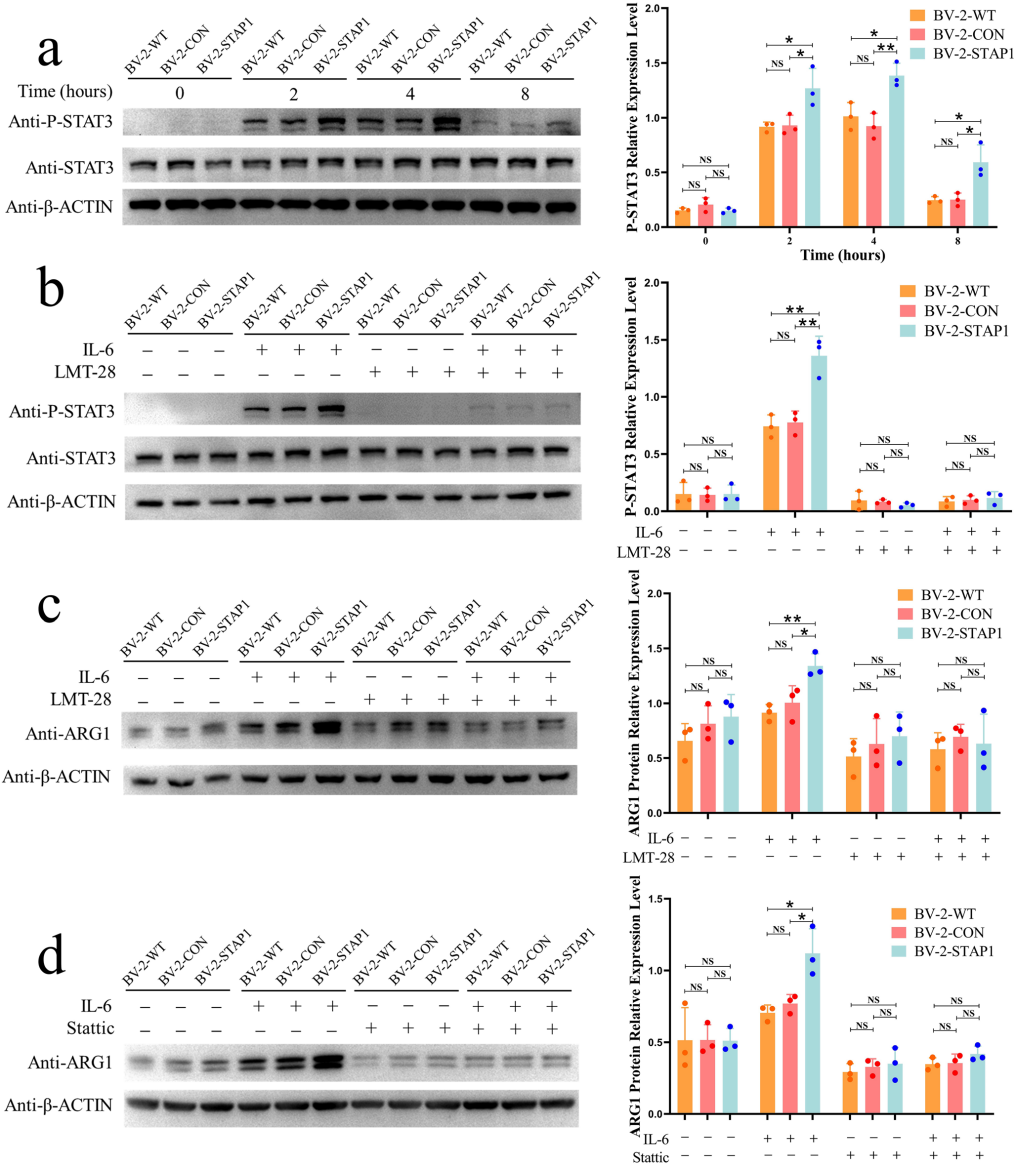
STAP1 overexpression promotes ARG1 expression via the IL-6/STAT3 pathway. **a** Phosphorylation levels of STAT3 at different time points (0 h, 2 h, 4 and 8 h) after stimulation with IL-6 (25ng/mL). Western blot analysis revealed higher phosphorylated STAT3 in BV-2-STAP1, which was most pronounced at 4 h. T-test. **b** Western blot analysis revealed high level of phosphorylated STAT3 in BV-2-STAP1 was decreased with LMT-28 (50 µM) treated. T-test. **c, d** Western blot analysis revealed high ARG1 protein level in BV-2-STAP1 was decreased with LMT-28 or Stattic (10 µM) treated. T-test. *P < 0.05, **P < 0.01

As a downstream target of IL-6, STAT3 is activated following IL-6 stimulation. To further confirm the pathway by which STAP1 promotes ARG1 expression, we measured STAT3 phosphorylation levels at different time points after IL-6 stimulation. STAP1 overexpression increased STAT3 phosphorylation, and the STAT3 phosphorylation level was most pronounced 4 h after stimulation with IL-6 (Fig. 3a). To further confirm this finding, we exposed the cells to the IL-6 inhibitor (LMT-28) and STAT3 inhibitor (Stattic) to reverse the effect of IL-6/STAT3 pathway. As expected, LMT-28 inhibited STAT3 phosphorylation and reversed the STAP1-induced high STAT3 phosphorylation levels (Fig. 3b). We also confirmed Stattic could inhibit STAT3 phosphorylation as previously reported [26] (Fig. S7).

IL-6 has long been known to promote ARG1 expression via the STAT3 pathway [25, 27]. In addition, phosphorylated STAT3 could bind with ARG1-promoter has been confirmed by CHIP-PCR [25, 28]. Same result was confirmed in this study (Fig. S8). Therefore, rescue experiment was conducted to validate whether LMT-28 or Stattic could reverse the STAP1-induced increase in ARG1 expression after IL-6 stimulation (Fig. 3c, d, S9). These results indicate that STAP1 promotes ARG1 expression via the IL-6/STAT3 pathway.

### STAP1 promotes microglia M2-like polarization by suppressing phagocytosis

**Fig. 4:**
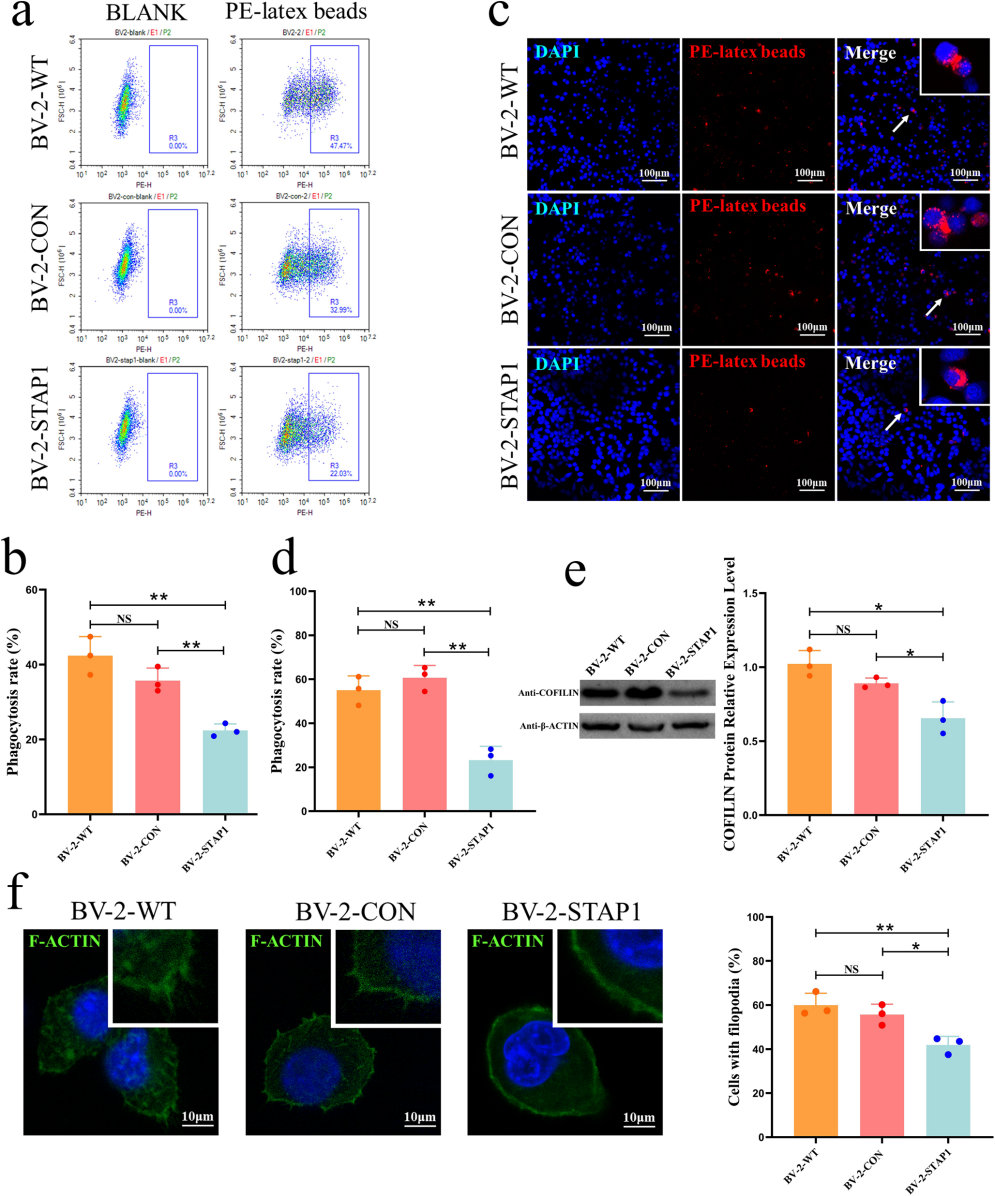
STAP1 overexpression promotes microglia M2-like polarisation by impairing the phagocytic function. **a**, **b** Flow cytometric analysis of the phagocytic function of BV2 cells. Less beads were engulfed in BV-2-STAP1 compared to the other cells. T-test. **c, d** Fluorescence analysis of the phagocytic function of BV2 cells. Less beads were engulfed in BV-2-STAP1 compared to the other cells. Scale bars, 100 μm. T-test. **e** Decreased protein level of cofilin was detected by Western blot. T-test. **f** Formation of filopodia was decreased in BV-2-STAP1 as detected by immunofluorescence. Scale bars, 10 μm. T-test. *P < 0.05, **P < 0.01

Decreased phagocytosis is a characteristic of M2-like polarisation [29]; therefore targeting phagocytic checkpoints and restoring macrophage function may play a critical role in cancer immunotherapy [30]. In the present study, cells were incubated with PE-latex beads for 3 h to detect the phagocytic function of BV2 cells. As shown in the flow cytometry plots, STAP1 suppressed phagocytic function of BV-2 cells (Fig. 4a, b), and similar results were obtained by direct visualisation by fluorescence microscopy (Fig. 4c, d).

A previous study has shown that cofilin knockdown inhibits microglial phagocytosis [31]. Moreover, filamentous actin (F-actin), a downstream protein of cofilin, can form filopodia, which is correlated with increased phagocytosis [32]. Therefore, we determined the expression level of cofilin and found that it was decreased in BV2-STAP1 cells (Fig. 4e). In addition, compared to the other groups, BV-2-STAP1 cells did not form filopodia obviously (Fig. 4f). These data indicate that STAP1 may reduce the expression level of cofilin and further impair the phagocytic function of BV-2 cells, which also indicates an M2-like phenotype.

### STAP1 in the TME of gliomas is a poor prognostic factor in vivo

**Fig. 5:**
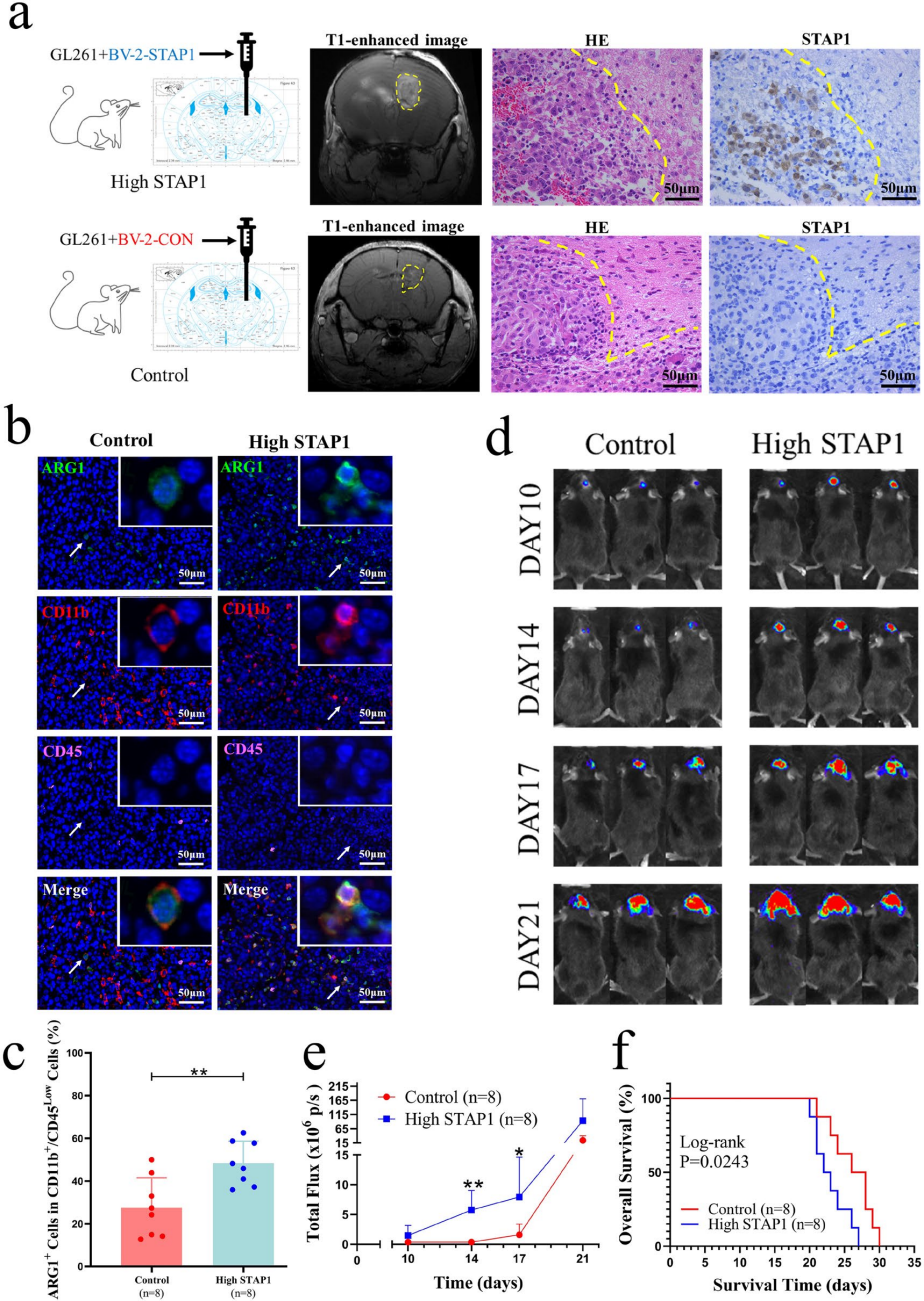
STAP1 in the glioma microenvironment is associated with poor prognosis. **a** Establishment of murine orthotopic glioma models that express high levels of STAP1 protein in TME. **b, c** Microglia express higher levels of ARG1 in the high STAP1 group. Scale bars, 50 μm. T-test. **d, e** Total photon flux was increased in the high STAP1 group especially on day 14 and 17. T-test. **f** Kaplan-Meier curves revealed the high STAP1 group have a shorter OS with significant difference in orthotopic glioma models. *P < 0.05, **P < 0.01

We have demonstrated that STAP1 promotes microglial M2-like polarisation and is associated with the degree of glioma malignancy. However, it remains unclear whether these results hold under in vivo conditions. Therefore, we mixed GL261 and BV-2-STAP1 cells and injected them into the murine right striatum to establish an orthotopic glioma model, in which the TME highly expresses STAP1 (Fig. 5a). Immunofluorescence analysis showed that STAP1 also induced microglial M2-like polarisation without exogeneous IL-6 stimulation, resulting in higher ARG1 expression levels in vivo (Fig. 5b, c). Glioma growth was monitored by bioluminescence imaging on given days (Fig. 5d); the high STAP1 group showed faster glioma growth, especially on days 14 and 17 (Fig. 5e). Similar results were observed in subcutaneous glioma mouse models. The volume of subcutaneous glioma was larger in the high-STAP1 group, especially on day 25 (Fig. S10). Furthermore, the Kaplan-Meier survival curves indicated that the OS of the control group was longer than that of the high-STAP1 group in orthotopic glioma models (Fig. 5f). These results indicate that, in vivo, STAP1 in the glioma microenvironment may promotes microglial M2-like polarisation and result in poor outcomes.

## Discussion

To date, there have been few studies on STAP1. STAP1, a downstream adaptor molecule of the B cell antigen receptor, may mediate plasma cell barrier reconstruction after intestinal transplantation [33]. However, no further study has substantiated these findings. Mutations in STAP1 have been reported to be associated with autosomal dominant hypercholesterolaemia [34]. Moreover, a previous study has revealed that STAP1 promotes haematological tumour progression [19]. Similarly, our findings also indicated that STAP1 may promote glioma progression. A previous study has shown that STAP1 may be associated with the activation of proinflammatory microglia and that the mRNA expression level of STAP1 is increased in proinflammatory cell models, stimulated by lipopolysaccharides [35]. Nevertheless, this study failed to prove that STAP1 regulate microglia toward an M1-like phenotype. Therefore, the evidence that STAP1 is a marker of the M1-like phenotype [36] is insufficient. The increased STAP1 expression in the previous study may be because STAP1 can attenuate proinflammatory responses to form negative feedback regulation. This notion is supported by our findings that STAP1 promotes microglial M2-like polarisation.

TAMs are generally have an M2-like phenotype for their tumour-promoting function [37]. The immunosuppressive M2-like phenotype is believed to be positive for ARG1 [38], a key regulator of arginine metabolism that transforms L-arginine to urea and L-ornithine, which are required for normal cell proliferation, collagen synthesis, tissue repair, and neuronal development [39]. ARG1 promotes tumour cell proliferation by enhancing the production of L-ornithine and putrescine. Moreover, ARG1 overexpression of impairs T-cell functions by downregulating the expression of the T-cell receptor (TCR) CD3zeta chain, a critical component of TCR signalling pathway [40]. In dendritic cells (DCs), phosphorylation of indoleamine 2,3-dioxygenase 1 (IDO1) and activation of the IDO downstream signalling pathway, which transforms DCs into an immunosuppressive phenotype, strictly depend on ARG1 and polyamines produced by ARG1 [41]. In the present study, we found STAP increased ARG1 expression level, which is a poor prognostic factor in tumours.

Generally, IL-4 can induce alternative macrophage activation by facilitating the phosphorylation of the transcription factor STAT6 [42]. However, in our study, no obvious increase in phosphorylated STAT6 and ARG1 levels was observed after IL-4 stimulation in BV-2-STAP1 cells (Fig. S11). In addition, IL-6 can also promote macrophages M2-like polarisation [43]. In glioblastoma, IL-6 secreted by endothelial cells induces macrophage M2-like polarisation by increasing ARG1 expression [27]. In TME, IL-6 promotes the proliferation and invasiveness of tumour cells and strongly inhibits anti-tumour immune responses [21]. STAT3, a downstream target of IL-6, suppresses the activation of immune cells and promotes the production of immunosuppressive factors [44]. Our previous studies also demonstrated that IL-6/STAT3 signalling plays an immunosuppressive role in GAMs [45]. These previous studies support the findings of the present study revealing that STAP1 promotes ARG1 expression, in response to IL-6 stimulation, indicating an M2-like phenotype, via the IL-6/STAT3 pathway. Although co-immunoprecipitation of STAP1 did not find STAP1 could combine with STAT3 directly (Fig. S12). A previous study demonstrated STAP1 could combine with SH-2 domain of STAT5a and maintain its phosphorylation, suggesting that STAP1 may affect the STAT protein family because they share the same SH-2 domain [19]. However, as a kind of adaptor protein, STAP1 lack enzymatic activity and direct effector function [46]. Thus, we speculate that STAP1 may affect IL-6/STAT3 pathway in an indirect manner and the further study will be conducted in the future.

Phagocytosis is another characteristic of microglia and macrophages [47]. M1-like polarisation promotes phagocytosis, whereas M2-like polarisation impairs it. A previous study has shown that alternatively activated macrophages (M2-like phenotype) impaired the phagocytic function of S.aureus in chronic rhinosinusitis [48]. Moreover, mesenchymal stem/stromal cells promote phagocytosis and M1-like polarisation of macrophages [49]. Glioma cells express high levels of CD47, which binds signal regulatory protein alpha of microglia, to release a “do not eat me” signal and suppress phagocytosis of microglia [50]. Therefore, targeting STAP1 to enhance phagocytosis and reverse the M2-like polarisation of microglia is a promising anti-tumour strategy for glioma treatment.

In conclusion, we found that STAP1 expression in glioma-associated microglia is positively correlated with the degree of malignancy and poor prognosis of glioma. Moreover, STAP1 may promote M2-like polarisation by increasing ARG1 expression and inhibiting microglial phagocytosis of microglia. Mechanistically, STAP1 enhances the phosphorylation of STAT3 under IL-6 stimulation. This study suggests STAP1 as a potential therapeutic target for glioma in the future.

## Electronic supplementary material

Below is the link to the electronic supplementary material.

Supplementary Material 1

## Data Availability

The data that support the findings of this study are available from the corresponding author on reasonable request to the corresponding author.

## References

[1] Ostrom QT, Cioffi G, Waite K, Kruchko C, Barnholtz-Sloan JS (2021) CBTRUS Statistical Report: primary brain and other Central Nervous System Tumors diagnosed in the United States in 2014–2018. Neurooncology 23. 10.1093/neuonc/noab200 10.1093/neuonc/noab200 PMC8491279 34608945

[2] Tan AC, Ashley DM, López GY, Malinzak M, Friedman HS, Khasraw M (2020). Management of glioblastoma: state of the art and future directions. CA Cancer J Clin.

[3] Agliardi G, Liuzzi AR, Hotblack A, De Feo D, Núñez N, Stowe CL, Friebel E, Nannini F, Rindlisbacher L, Roberts TA, Ramasawmy R, Williams IP, Siow BM, Lythgoe MF, Kalber TL, Quezada SA, Pule MA, Tugues S, Straathof K, Becher B (2021). Intratumoral IL-12 delivery empowers CAR-T cell immunotherapy in a pre-clinical model of glioblastoma. Nat Commun.

[4] Martínez-Reyes I, Chandel NS (2021). Cancer metabolism: looking forward. Nat Rev Cancer.

[5] Hinshaw DC, Shevde LA (2019). The Tumor Microenvironment innately modulates Cancer Progression. Cancer Res.

[6] Pittet MJ, Michielin O, Migliorini D (2022). Clinical relevance of tumour-associated macrophages. Nat Rev Clin Oncol.

[7] Quail DF, Joyce JA (2017). The Microenvironmental Landscape of Brain Tumors. Cancer Cell.

[8] Müller A, Brandenburg S, Turkowski K, Müller S, Vajkoczy P (2015). Resident microglia, and not peripheral macrophages, are the main source of brain tumor mononuclear cells. Int J Cancer.

[9] Brandenburg S, Müller A, Turkowski K, Radev YT, Rot S, Schmidt C, Bungert AD, Acker G, Schorr A, Hippe A, Miller K, Heppner FL, Homey B, Vajkoczy P (2016). Resident microglia rather than peripheral macrophages promote vascularization in brain tumors and are source of alternative pro-angiogenic factors. Acta Neuropathol.

[10] Wynn TA, Chawla A, Pollard JW (2013). Macrophage biology in development, homeostasis and disease. Nature.

[11] Gordon S (2003). Alternative activation of macrophages. Nat Rev Immunol.

[12] Gao X, Li S, Ding F, Liu X, Wu Y, Li J, Feng J, Zhu X, Zhang C (2021). A virus-mimicking nucleic acid Nanogel Reprograms Microglia and Macrophages for Glioblastoma Therapy. Adv Mater.

[13] Hambardzumyan D, Gutmann DH, Kettenmann H (2016). The role of microglia and macrophages in glioma maintenance and progression. Nat Neurosci.

[14] Venteicher AS, Tirosh I, Hebert C, Yizhak K, Neftel C, Filbin MG, Hovestadt V, Escalante LE, Shaw ML, Rodman C, Gillespie SM, Dionne D, Luo CC, Ravichandran H, Mylvaganam R, Mount C, Onozato ML, Nahed BV, Wakimoto H, Curry WT, Iafrate AJ, Rivera MN, Frosch MP, Golub TR, Brastianos PK, Getz G, Patel AP, Monje M, Cahill DP, Rozenblatt-Rosen O, Louis DN, Bernstein BE, Regev A, Suvà ML (2017). Decoupling genetics, lineages, and microenvironment in IDH-mutant gliomas by single-cell RNA-seq. Sci 355.

[15] Klemm F, Maas RR, Bowman RL, Kornete M, Soukup K, Nassiri S, Brouland J-P, Iacobuzio-Donahue CA, Brennan C, Tabar V, Gutin PH, Daniel RT, Hegi ME, Joyce JA (2020) Interrogation of the Microenvironmental Landscape in Brain Tumors reveals Disease-Specific alterations of Immune cells. Cell 181. 10.1016/j.cell.2020.05.007 10.1016/j.cell.2020.05.007 PMC8558904 32470396

[16] Andersen BM, Faust Akl C, Wheeler MA, Chiocca EA, Reardon DA, Quintana FJ (2021). Glial and myeloid heterogeneity in the brain tumour microenvironment. Nat Rev Cancer.

[17] Shemer A, Grozovski J, Tay TL, Tao J, Volaski A, Süß P, Ardura-Fabregat A, Gross-Vered M, Kim J-S, David E, Chappell-Maor L, Thielecke L, Glass CK, Cornils K, Prinz M, Jung S (2018). Engrafted parenchymal brain macrophages differ from microglia in transcriptome, chromatin landscape and response to challenge. Nat Commun.

[18] Ohya K, Kajigaya S, Kitanaka A, Yoshida K, Miyazato A, Yamashita Y, Yamanaka T, Ikeda U, Shimada K, Ozawa K, Mano H (1999). Molecular cloning of a docking protein, BRDG1, that acts downstream of the Tec tyrosine kinase. Proc Natl Acad Sci USA.

[19] Toda J, Ichii M, Oritani K, Shibayama H, Tanimura A, Saito H, Yokota T, Motooka D, Okuzaki D, Kitai Y, Muromoto R, Kashiwakura J-I, Matsuda T, Hosen N, Kanakura Y (2020). Signal-transducing adapter protein-1 is required for maintenance of leukemic stem cells in CML. Oncogene.

[20] Sekine Y, Ikeda O, Mizushima A, Ueno Y, Muromoto R, Yoshimura A, Kanakura Y, Oritani K, Matsuda T (2012). STAP-2 interacts with and modulates BCR-ABL-mediated tumorigenesis. Oncogene.

[21] Johnson DE, O’Keefe RA, Grandis JR (2018). Targeting the IL-6/JAK/STAT3 signalling axis in cancer. Nat Rev Clin Oncol.

[22] Chen D, Li G, Ji C, Lu Q, Qi Y, Tang C, Xiong J, Hu J, Yasar FBA, Zhang Y, Hoon DSB, Yao Y, Zhou L (2020) Enhanced B7-H4 expression in gliomas with low PD-L1 expression identifies super-cold tumors. J Immunother Cancer 8. 10.1136/jitc-2019-000154 10.1136/jitc-2019-000154 PMC7253052 32457124

[23] Vinnakota K, Hu F, Ku M-C, Georgieva PB, Szulzewsky F, Pohlmann A, Waiczies S, Waiczies H, Niendorf T, Lehnardt S, Hanisch U-K, Synowitz M, Markovic D, Wolf SA, Glass R, Kettenmann H (2013). Toll-like receptor 2 mediates microglia/brain macrophage MT1-MMP expression and glioma expansion. Neurooncology.

[24] Prinz M, Masuda T, Wheeler MA, Quintana FJ (2021). Microglia and Central Nervous System-Associated Macrophages-From origin to Disease Modulation. Annu Rev Immunol.

[25] Jin Y, Kang Y, Wang M, Wu B, Su B, Yin H, Tang Y, Li Q, Wei W, Mei Q, Hu G, Lukacs-Kornek V, Li J, Wu K, Yuan X, Wang W (2022). Targeting polarized phenotype of microglia via IL6/JAK2/STAT3 signaling to reduce NSCLC brain metastasis. Signal Transduct Target Ther.

[26] Yamamoto K, Uda A, Mukai A, Yamashita K, Kume M, Makimoto H, Bito T, Nishigori C, Hirano T, Hirai M (2013). Everolimus-induced human keratinocytes toxicity is mediated by STAT3 inhibition. J Exp Clin Cancer Res.

[27] Wang Q, He Z, Huang M, Liu T, Wang Y, Xu H, Duan H, Ma P, Zhang L, Zamvil SS, Hidalgo J, Zhang Z, O’Rourke DM, Dahmane N, Brem S, Mou Y, Gong Y, Fan Y (2018). Vascular niche IL-6 induces alternative macrophage activation in glioblastoma through HIF-2α. Nat Commun.

[28] Vasquez-Dunddel D, Pan F, Zeng Q, Gorbounov M, Albesiano E, Fu J, Blosser RL, Tam AJ, Bruno T, Zhang H, Pardoll D, Kim Y (2013). STAT3 regulates arginase-I in myeloid-derived suppressor cells from cancer patients. J Clin Invest.

[29] Wu A, Wei J, Kong L-Y, Wang Y, Priebe W, Qiao W, Sawaya R, Heimberger AB (2010). Glioma cancer stem cells induce immunosuppressive macrophages/microglia. Neurooncology.

[30] Feng M, Jiang W, Kim BYS, Zhang CC, Fu Y-X, Weissman IL (2019). Phagocytosis checkpoints as new targets for cancer immunotherapy. Nat Rev Cancer.

[31] Alhadidi Q, Shah ZA (2018). Cofilin mediates LPS-Induced Microglial Cell activation and Associated Neurotoxicity through activation of NF-κB and JAK-STAT pathway. Mol Neurobiol.

[32] Geng J, Shi Y, Zhang J, Yang B, Wang P, Yuan W, Zhao H, Li J, Qin F, Hong L, Xie C, Deng X, Sun Y, Wu C, Chen L, Zhou D (2021). TLR4 signalling via Piezo1 engages and enhances the macrophage mediated host response during bacterial infection. Nat Commun.

[33] Ningappa M, Ashokkumar C, Ranganathan S, Schmitt L, Higgs BW, Sun Q, Branca M, Mazariegos G, Zeevi A, Abu-Elmagd K, Squires R, Rudolph J, Alissa F, Hakonarson H, Sindhi R (2012). Mucosal plasma cell barrier disruption during intestine transplant rejection. Transplantation.

[34] Fouchier SW, Dallinga-Thie GM, Meijers JCM, Zelcer N, Kastelein JJP, Defesche JC, Hovingh GK (2014). Mutations in STAP1 are associated with autosomal dominant hypercholesterolemia. Circ Res.

[35] Stoecker K, Weigelt K, Ebert S, Karlstetter M, Walczak Y, Langmann T (2009). Induction of STAP-1 promotes neurotoxic activation of microglia. Biochem Biophys Res Commun.

[36] Ji W-H, Li D-D, Wei D-P, Gu A-Q, Yang Y, Peng J-P (2021). Cytochrome P450 26A1 modulates the polarization of Uterine Macrophages during the Peri-Implantation Period. Front Immunol.

[37] Mehla K, Singh PK (2019). Metabolic regulation of macrophage polarization in Cancer. Trends Cancer.

[38] Arlauckas SP, Garren SB, Garris CS, Kohler RH, Oh J, Pittet MJ, Weissleder R (2018). Arg1 expression defines immunosuppressive subsets of tumor-associated macrophages. Theranostics.

[39] Caldwell RW, Rodriguez PC, Toque HA, Narayanan SP, Caldwell RB (2018). Arginase: a multifaceted enzyme important in Health and Disease. Physiol Rev.

[40] Rodriguez PC, Quiceno DG, Zabaleta J, Ortiz B, Zea AH, Piazuelo MB, Delgado A, Correa P, Brayer J, Sotomayor EM, Antonia S, Ochoa JB, Ochoa AC (2004). Arginase I production in the tumor microenvironment by mature myeloid cells inhibits T-cell receptor expression and antigen-specific T-cell responses. Cancer Res.

[41] Mondanelli G, Bianchi R, Pallotta MT, Orabona C, Albini E, Iacono A, Belladonna ML, Vacca C, Fallarino F, Macchiarulo A, Ugel S, Bronte V, Gevi F, Zolla L, Verhaar A, Peppelenbosch M, Mazza EMC, Bicciato S, Laouar Y, Santambrogio L, Puccetti P, Volpi C, Grohmann U (2017). A relay pathway between Arginine and Tryptophan Metabolism confers immunosuppressive Properties on dendritic cells. Immunity.

[42] Czimmerer Z, Daniel B, Horvath A, Rückerl D, Nagy G, Kiss M, Peloquin M, Budai MM, Cuaranta-Monroy I, Simandi Z, Steiner L, Nagy B, Poliska S, Banko C, Bacso Z, Schulman IG, Sauer S, Deleuze J-F, Allen JE, Benko S, Nagy L (2018) The transcription factor STAT6 mediates direct repression of inflammatory enhancers and limits activation of alternatively polarized macrophages. 10.1016/j.immuni.2017.12.010. Immunity 48 10.1016/j.immuni.2017.12.010 PMC5772169 29343442

[43] Weng Y-S, Tseng H-Y, Chen Y-A, Shen P-C, Al Haq AT, Chen L-M, Tung Y-C, Hsu H-L (2019). MCT-1/miR-34a/IL-6/IL-6R signaling axis promotes EMT progression, cancer stemness and M2 macrophage polarization in triple-negative breast cancer. Mol Cancer.

[44] Yu H, Kortylewski M, Pardoll D (2007). Crosstalk between cancer and immune cells: role of STAT3 in the tumour microenvironment. Nat Rev Immunol.

[45] Yao Y, Ye H, Qi Z, Mo L, Yue Q, Baral A, Hoon DSB, Vera JC, Heiss JD, Chen CC, Zhang J, Jin K, Wang Y, Zang X, Mao Y, Zhou L (2016). B7-H4(B7x)-Mediated cross-talk between glioma-initiating cells and macrophages via the IL6/JAK/STAT3 pathway lead to poor prognosis in Glioma Patients. Clin Cancer Res.

[46] Jordan MS, Singer AL, Koretzky GA (2003). Adaptors as central mediators of signal transduction in immune cells. Nat Immunol.

[47] Zhang C, Zhou Y, Gao Y, Zhu Z, Zeng X, Liang W, Sun S, Chen X, Wang H (2022). Radiated glioblastoma cell-derived exosomal circ_0012381 induce M2 polarization of microglia to promote the growth of glioblastoma by CCL2/CCR2 axis. J Transl Med.

[48] Krysko O, Holtappels G, Zhang N, Kubica M, Deswarte K, Derycke L, Claeys S, Hammad H, Brusselle GG, Vandenabeele P, Krysko DV, Bachert C (2011). Alternatively activated macrophages and impaired phagocytosis of S. aureus in chronic rhinosinusitis. Allergy.

[49] Rabani R, Volchuk A, Jerkic M, Ormesher L, Garces-Ramirez L, Canton J, Masterson C, Gagnon S, Tatham KC, Marshall J, Grinstein S, Laffey JG, Szaszi K, Curley GF (2018) Mesenchymal stem cells enhance NOX2-dependent reactive oxygen species production and bacterial killing in macrophages during sepsis. Eur Respir J 51. 10.1183/13993003.02021-2017 10.1183/13993003.02021-2017 29519920

[50] Hutter G, Theruvath J, Graef CM, Zhang M, Schoen MK, Manz EM, Bennett ML, Olson A, Azad TD, Sinha R, Chan C, Assad Kahn S, Gholamin S, Wilson C, Grant G, He J, Weissman IL, Mitra SS, Cheshier SH (2019) Microglia are effector cells of CD47-SIRPα antiphagocytic axis disruption against glioblastoma. Proc Natl Acad Sci USA 116. 10.1073/pnas.1721434116 10.1073/pnas.1721434116 PMC6338872 30602457

