# Supplementary material for: Signal-transducing adaptor protein 1 (STAP1) in microglia promotes the malignant progression of glioma
Source: J Neurooncol. 2023 Jul 18;164(1):127–39. doi: 10.1007/s11060-023-04390-8 (PMC10462508; doi:10.1007/s11060-023-04390-8)
Supplement: Supplementary file 1 — Supplementary Material 1 [file 11060_2023_4390_MOESM1_ESM.docx]

Signal-transducing adaptor protein 1 (STAP1) in microglia promotes the malignant progression of glioma

Xinyu Yang^1,2,3,4,5^, Chunxia Ji^1,2,3,4,5^, Ying Qi^1,2,3,4,5^, Jianhan Huang^1,2,3,4,5^, Lang Hu^1,2,3,4,5^, Yuan Zhou^1,2,3,4,5^, Liping Zou^6^, Yi Xia^6^, Feng Tan^7^, Yu Yao^1,2,3,4,5^, Di Chen^1,2,3,4,5^

1 Department of Neurosurgery, Huashan Hospital, Shanghai Medical College, Fudan University, Shanghai, China

2 National Center for Neurological Disorders, Shanghai, China

3 Shanghai Key Laboratory of Brain Function and Restoration and Neural Regeneration, Shanghai, China

4 Immunology Laboratory, Neurosurgical Institute of Fudan University, Shanghai, China

5 Shanghai Clinical Medical Center of Neurosurgery, Shanghai, China

6 Department of Pathology, Huashan Hospital, Fudan University, Shanghai, China

7 School of Basic Medical Sciences, Wenzhou Medical University, Wenzhou, Zhejiang, China

Di Chen: major corresponding author; email: dichen18@fudan.edu.cn

YY: co-corresponding author; email: yu_yao@fudan.edu.cn

This manuscript is submitted for “Journal of Neuro-oncology”


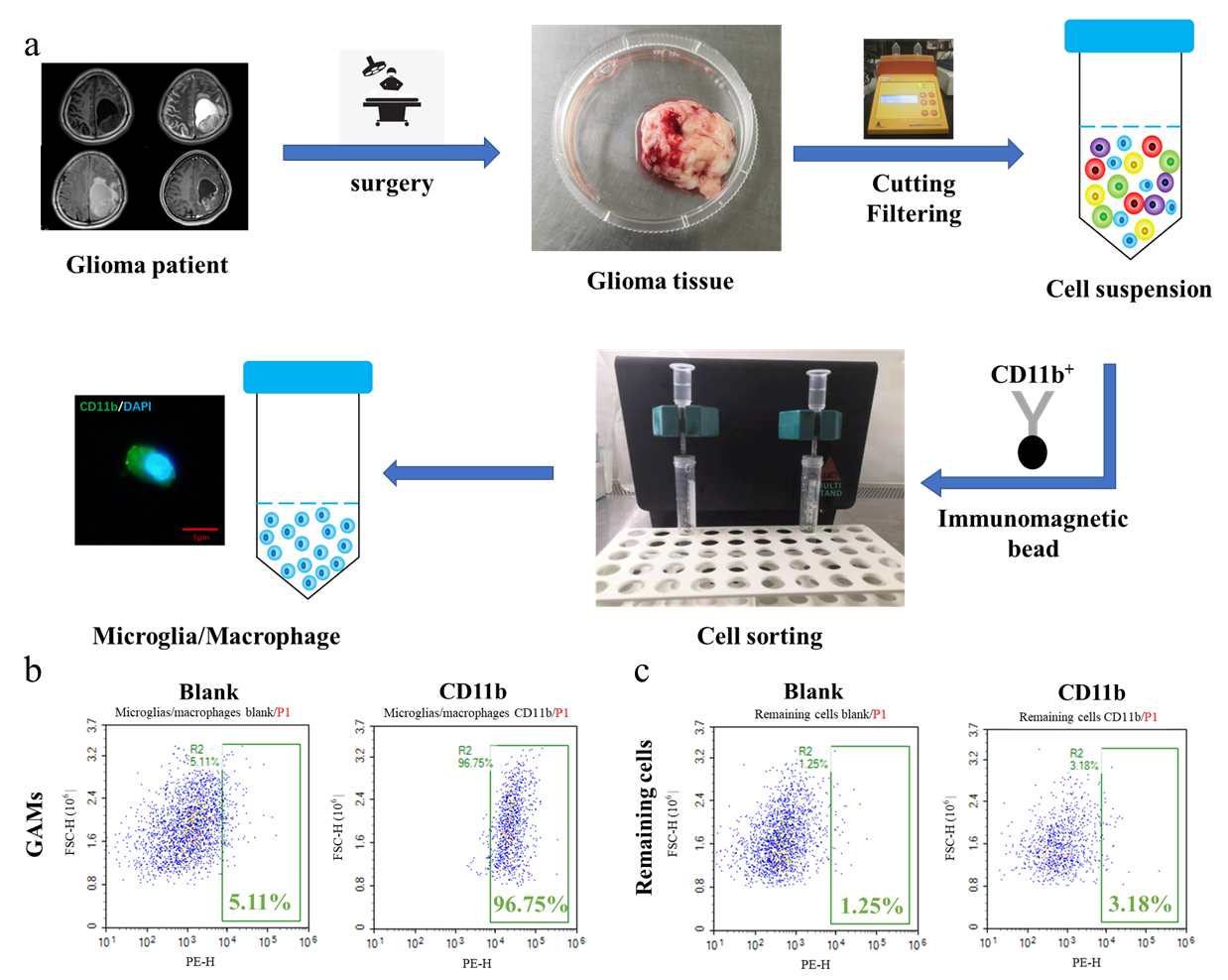


**Fig. S1 GAMs isolation. a** The process of isolating GAMs from glioma tissue. **b** Isolated cells (GAMs) highly express CD11b. **c** Residual cells contain a small number of GAMs.


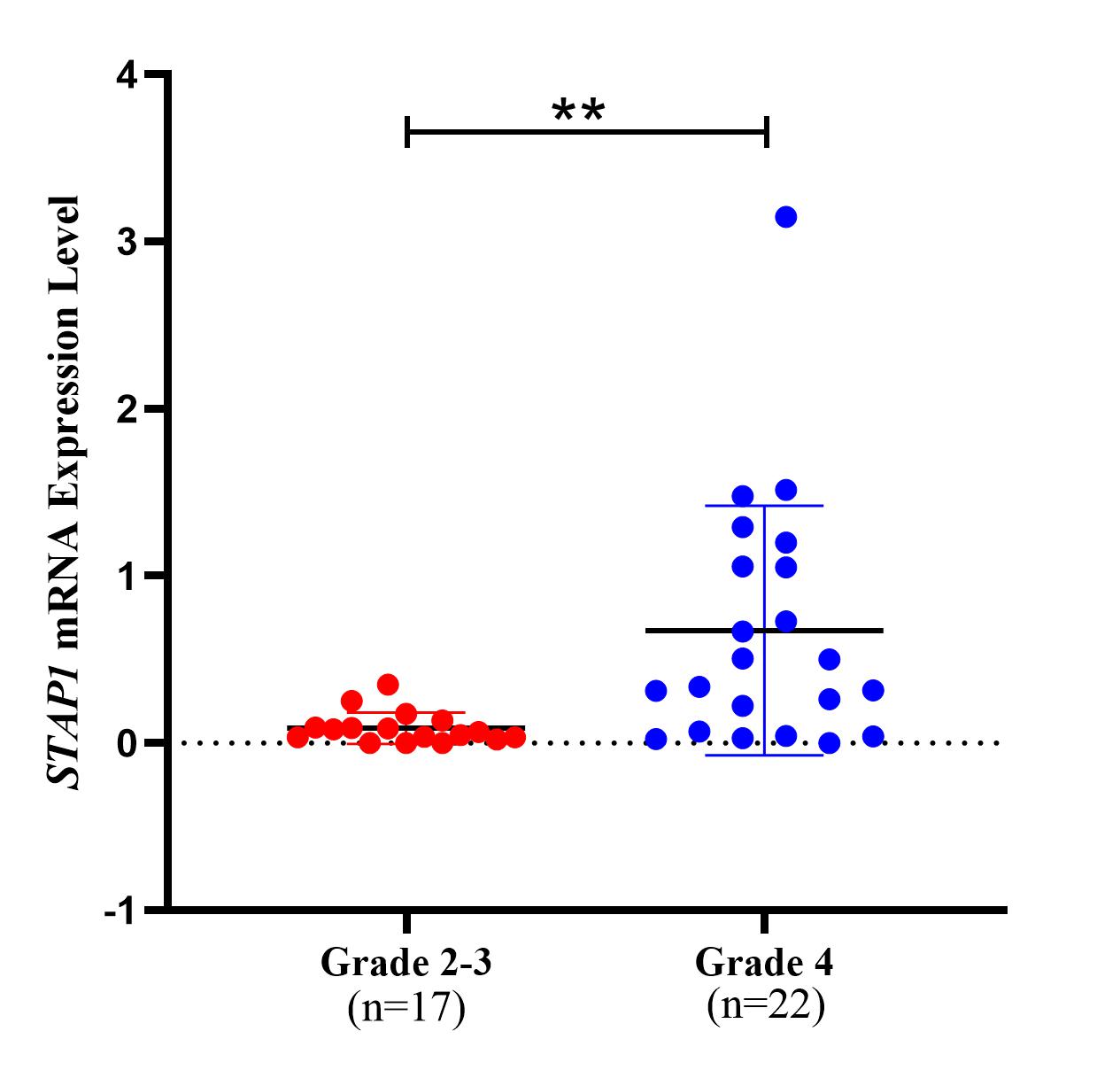


**Fig. S2 STAP1 was found by transcriptome sequencing of GAMs.** STAP1 mRNA was higher in GAMs of grade 4 glioma than of grade 2-3 glioma.


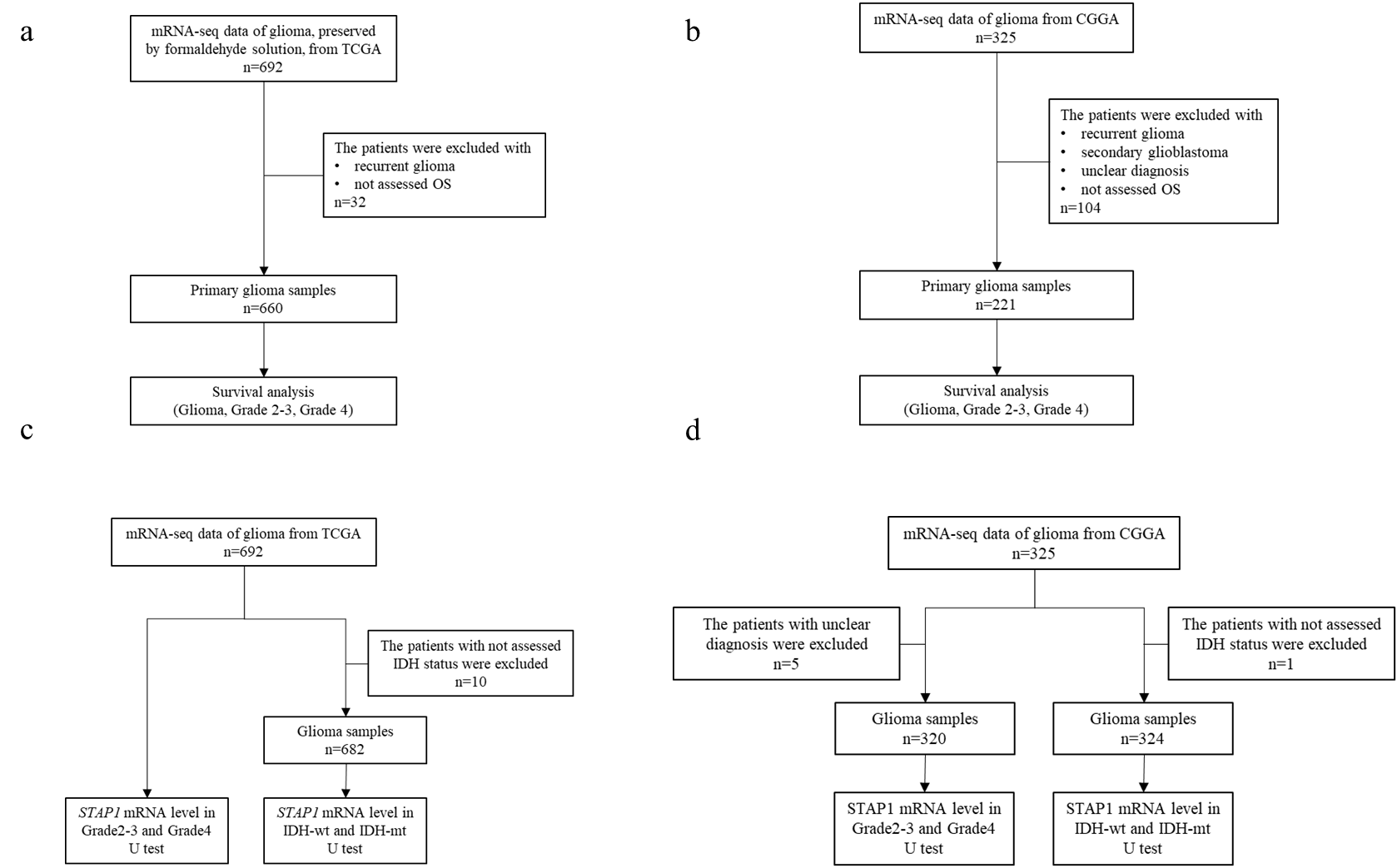


**Fig. S3 Inclusion criteria and exclusion criteria of TCGA data and CGGA data. a, b** Inclusion criteria and exclusion criteria of survival analysis in TCGA and CGGA. **c, d** Inclusion criteria and exclusion criteria of STAP1 mRNA level in TCGA and CGGA.

**Fig. S4 Survival analysis of patients with glioma in TCGA datasets and CGGA datasets. a, b** The low-STAP1 group has higher OS in patients with glioma and in patients with grade 2-3 glioma. **c** In patients with grade 4 glioma, the low-STAP1 group tends to live longer with no statistically significant differences.
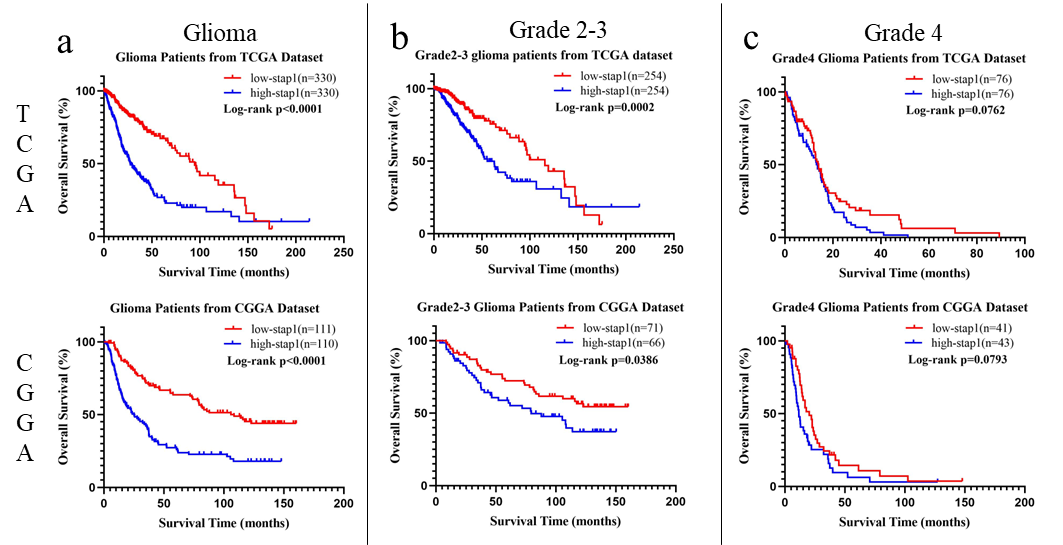


| **Number** | **Pathological grade** | **IHC** | **WB** | **qRT-PCR** |
| --- | --- | --- | --- | --- |
| 2020-001 | 3 | **√** |  | **√** |
| 2020-002 | 2 |  |  | **√** |
| 2020-003 | 2 | **√** |  | **√** |
| 2020-004 | 2 | **√** |  | **√** |
| 2020-005 | 4 | **√** | **√** | **√** |
| 2021-001 | 3 | **√** | **√** | **√** |
| 2021-002 | 4 | **√** | **√** | **√** |
| 2021-003 | 2 | **√** | **√** | **√** |
| 2021-004 | 3 | **√** | **√** | **√** |
| 2021-005 | 4 | **√** | **√** | **√** |
| 2021-006 | 2 | **√** | **√** | **√** |
| 2021-007 | 2 |  | **√** | **√** |
| 2021-008 | 4 | **√** | **√** | **√** |
| 2021-009 | 4 | **√** | **√** | **√** |
| 2021-010 | 2 | **√** | **√** | **√** |
| 2021-011 | 4 | **√** | **√** | **√** |
| 2021-012 | 2 | **√** | **√** | **√** |
| 2021-013 | 3 | **√** | **√** |  |
| 2021-014 | 3 |  | **√** |  |
| 2021-015 | 4 | **√** | **√** | **√** |
| 2021-016 | 3 | **√** | **√** | **√** |
| 2021-017 | 4 | **√** | **√** | **√** |
| 2021-018 | 2 | **√** | **√** | **√** |
| 2021-019 | 4 | **√** | **√** | **√** |
| 2021-020 | 4 | **√** | **√** | **√** |
| 2021-021 | 2 | **√** | **√** |  |
| 2021-022 | 2 |  | **√** |  |
| 2021-023 | 4 | **√** | **√** | **√** |
| 2021-024 | 4 | **√** | **√** | **√** |
| 2021-025 | 4 | **√** | **√** | **√** |

**Table S1. Glioma samples for immunohistochemistry and their GAMs for** **western blot and qRT-PCR.**


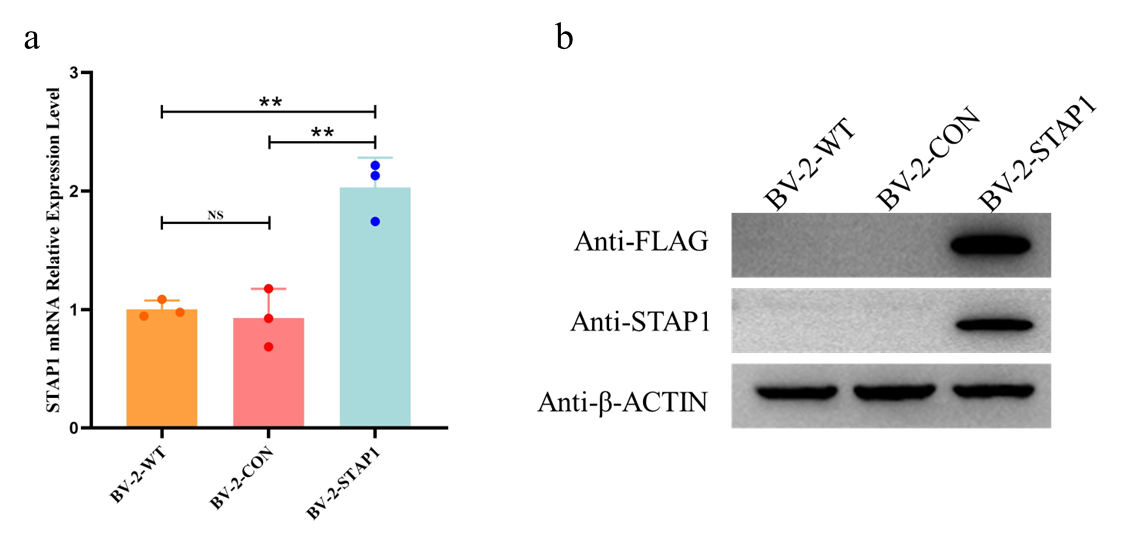


**Fig. S5 Establishment of high STAP1 microglia models.** **a** *Stap1* mRNA was higher in BV-2-STAP1. **b** STAP1 protein was higher in BV-2-STAP1.

| **Target** | **Species** | **Forward Primer** | **Reverse Primer** |
| --- | --- | --- | --- |
| *STAP1* | Human | CGGTCAGGATACCGGGAGTA | GCTCAGTAAGGCATGTGAGGT |
| *GAPDH* | Human | GAAGGTGAAGGTCGGAGTC | GAAGATGGTGATGGGATTTC |
| *Stap1* | Murine | GGAGGGGCTTCATTCTTACA | TGCCCAGGTAGAAGTGACA |
| *Arg1* | Murine | AGGAAAGCTGGTCTGCTGGAA | AGATGCTTCCAACTGCCAGAC |
| *Gapdh* | Murine | TGACCTCAACTACATGGTCTACA | CTTCCCATTCTCGGCCTTG |

**Table S2. Primers of qRT-PCR.**

|  | **Forward Primer** | **Reverse Primer** |
| --- | --- | --- |
| Primer 1 | CTCTCCACCGGCCGTAAC | GGTGTGAACTGGACGGATGA |
| Primer 2 | GAGCTCATGCTCTCTCTGGG | GTTACGGCCGGTGGAGAG |
| Primer 3 | CTGCTGCTGCATGTGCTC | GCGGAGCCAGTTGTTGGATA |

**Table S3. Primers of CHIP-PCR.**


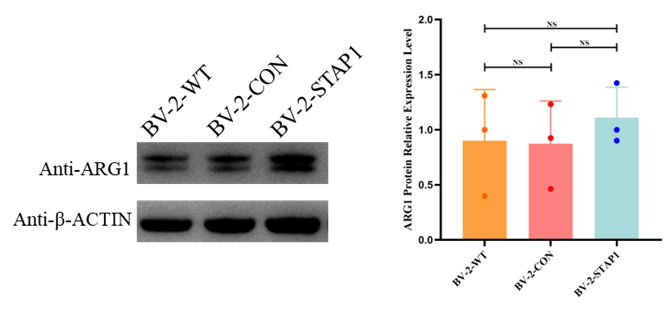


**Fig. S6 ARG1 expression level.** ARG1 was increased slightly in BV-2-STAP1 with no significance.


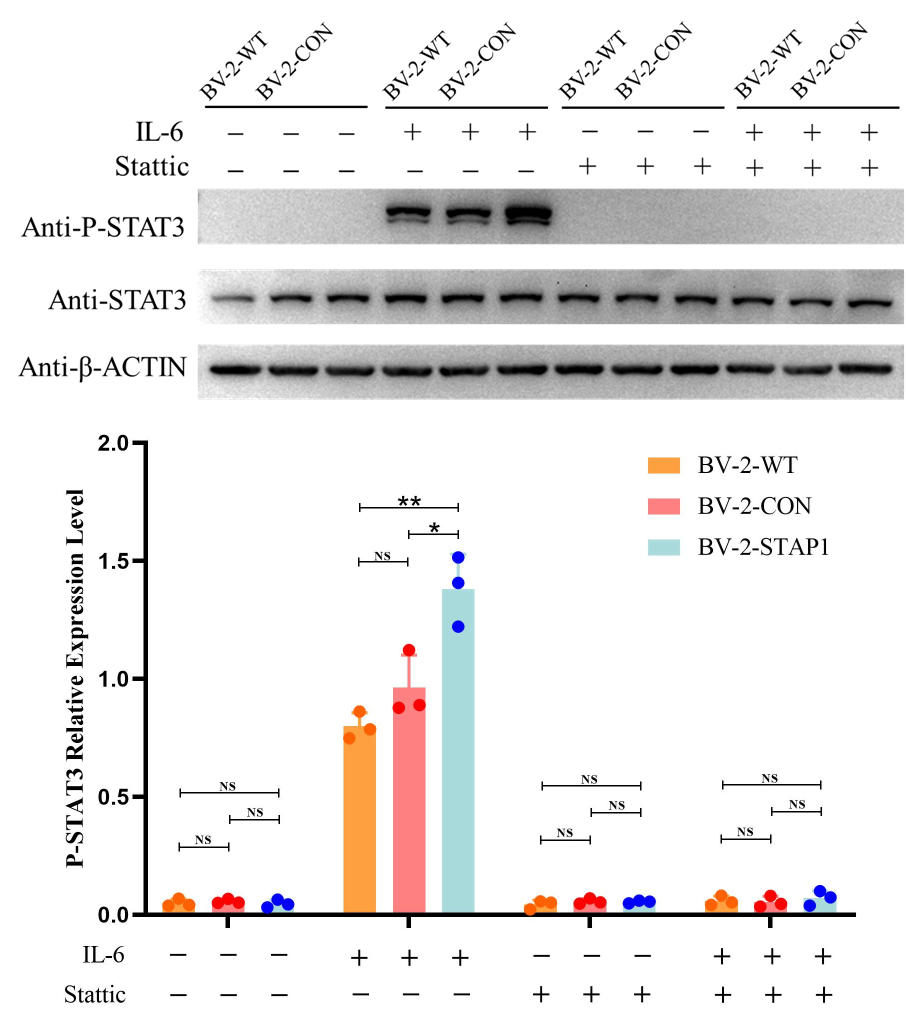


**Fig. S7 Phosphorylation level of STAT3.** Western blot analysis revealed Stattic could inhibit high level of phosphorylated STAT3 stimulated by IL-6.


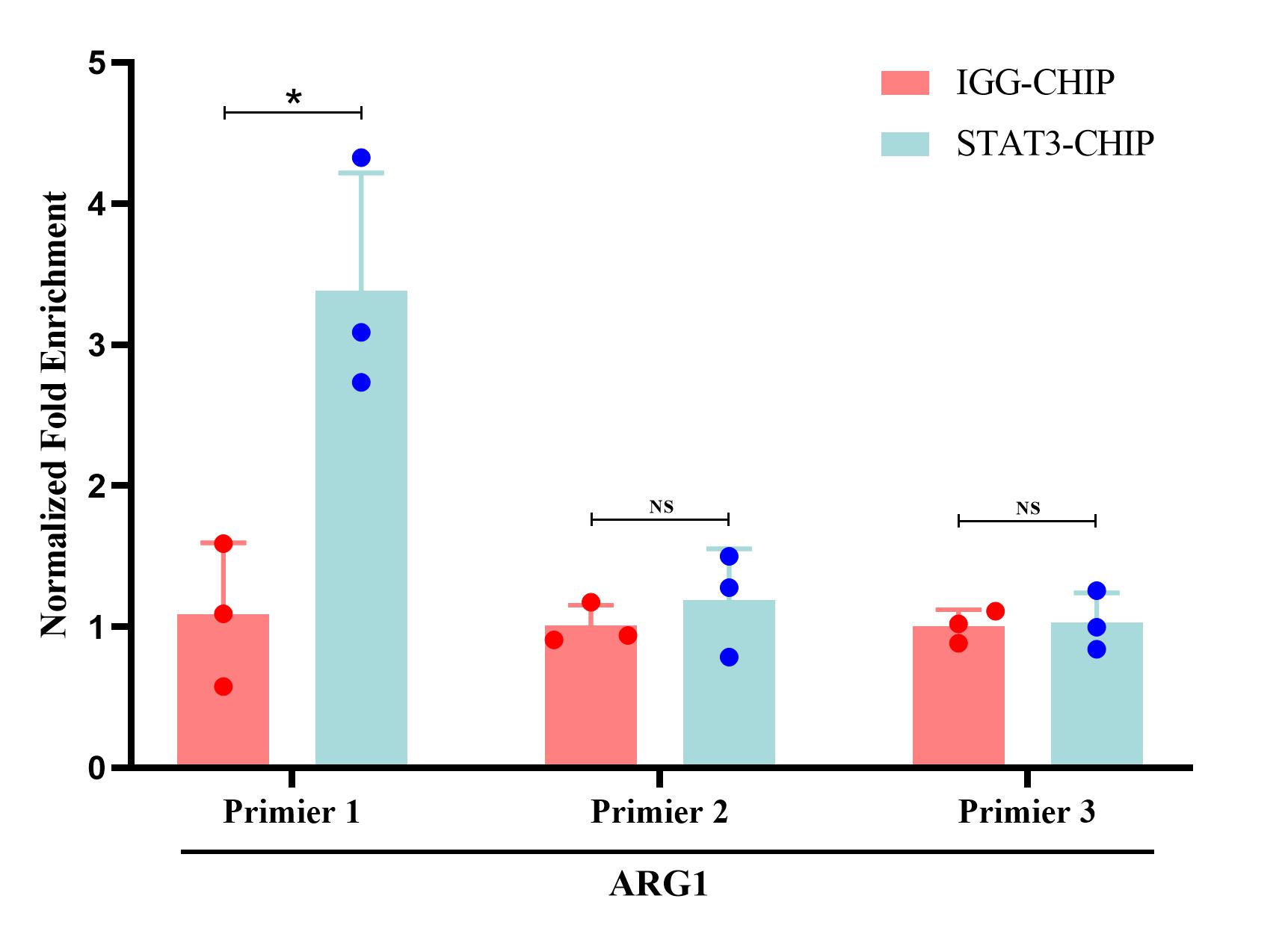


**Fig. S8** **STAT3 binds to the promoter region of ARG1.** CHIP-PCR analysis showed phosphorylated STAT3 could activate ARG1-promoter.

**
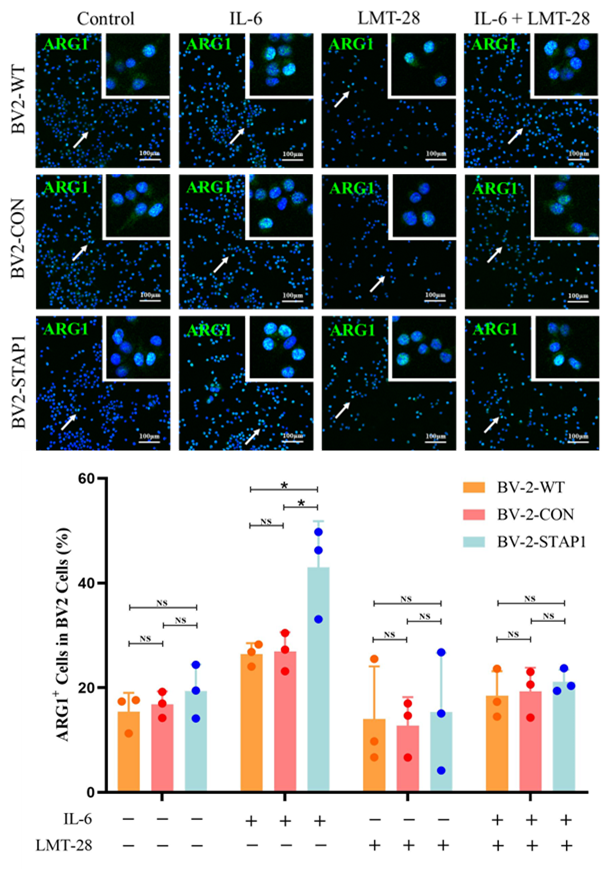
**

**Fig. S9 expression level of ARG1. Immunofluorescence analysis revealed high ARG1 protein level in BV-2-STAP1 was decreased with LMT-28 treated.**


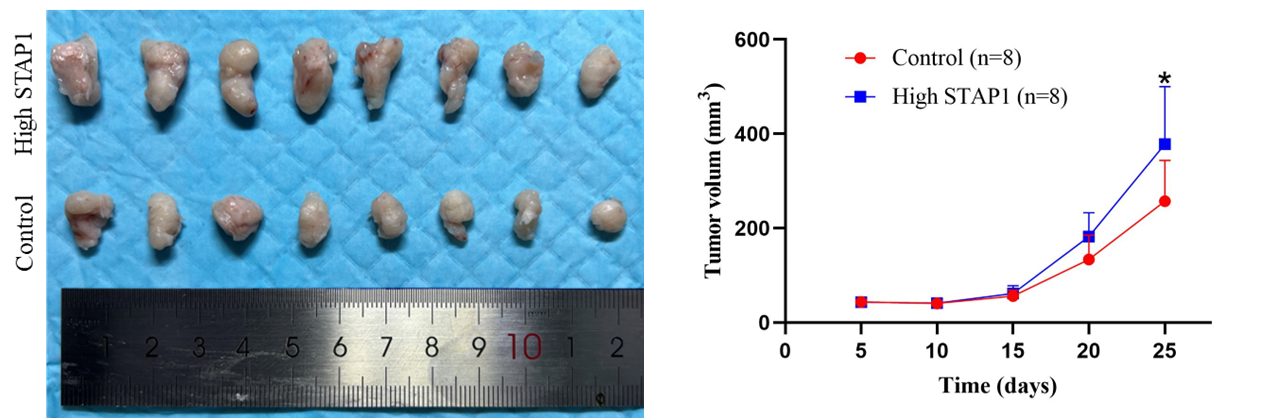


**Fig. S10 STAP1 promotes growth of glioma in subcutaneous glioma mouse models.** Subcutaneous glioma grew faster in high STAP1 group especially on day 25.


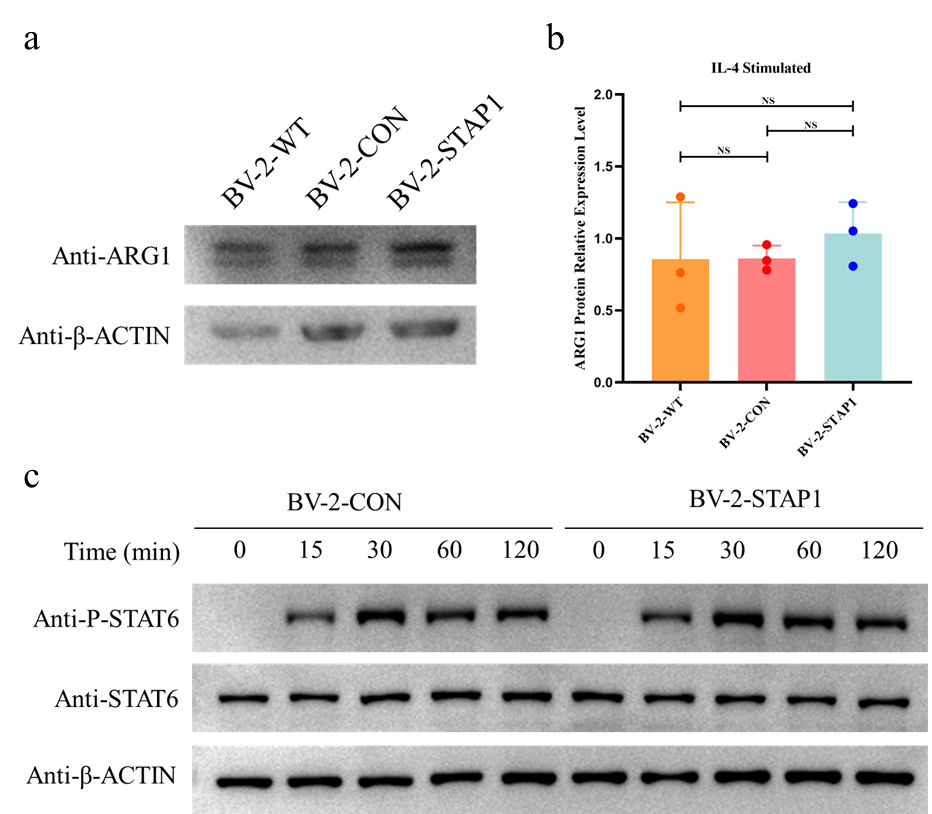


**Fig. S11 ARG1 expression level after IL-4 stimulation. a, b** No differences of ARG1 protein level could be found after IL-4 stimulation. c No differences of phosphorylated STAT6 level could be found after IL-4 stimulation.


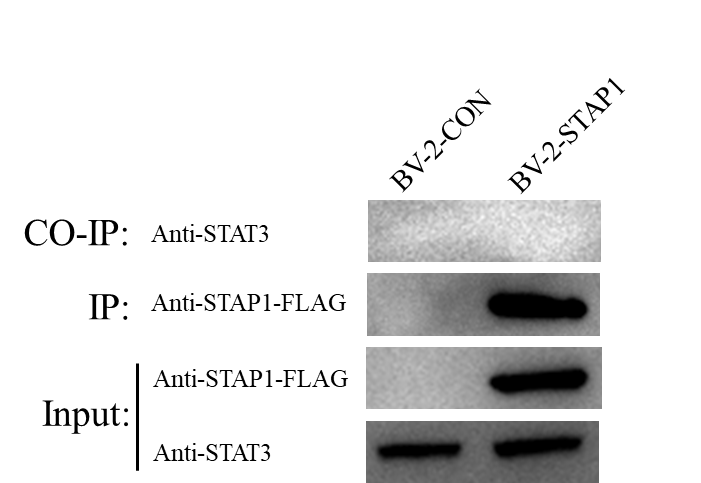


**Fig. S12 Co-immunoprecipitation of STAP1.** Western blot analysis revealed STAP1 may not bind with STAT3 directly.

**Supplementary Materials and methods**

**mRNA Isolation and qRT-PCR**

mRNA was isolated using the Total RNA Extraction Kit (R1200, Solarbio) and reverse-transcribed into cDNA by qRT-PCR Kit (DBI-2000, DBI Bioscience). Gene amplification was performed using Sybrgreen qPCR mix (DBI-2043, DBI Bioscience) under the following PCR conditions: 95 ℃ for 10 min, 95 ℃ for 15 s, 59 ℃ for 1 min, and 72 ℃ for 30 s for 40 cycles, 45 ℃ for 1 min. We measured transcripts of STAP1 in human GAMs. For BV-2, BV-2-CON and BV-2-STAP1 cells, we measured the transcript levels of Stap1 and Arg1. All transcripts were normalised to GAPDH. The primer sequences used are listed in Table. S2.

**RNA-Sequencing (RNA-seq)**

RNA-seq of GAMs was performed by Gemple Biotechnology (Shanghai, China). RNA-seq of BV-2-STAP1 and BV-2-CON was carried out by Mingma Technologies (Shanghai, China) using a second-generation sequencing technique (Illumina Novaseq 6000 platform). Raw reads were quality-controlled using FastQC (v0.11.9) (Andrews, 2010). Gene expression was quantified using RSEM (v1.2.29). Differential analysis was performed using the edgeR (v3.28.1).

**Western Blotting**

Cells were lysed in RIPA lysis buffer (P0013B, Beyotime) supplemented with protease inhibitors (BL507A, Biosharp) and phosphatase inhibitors (BL615A, Biosharp). Cell lysates were then added to the loading buffer (P1040, Solarbio) and boiled for 10 min at 100°C. Samples were loaded onto sodium dodecyl sulphate-polyacrylamide gel, electrophoresed, transferred onto nitrocellulose membranes, incubated with appropriate antibodies and finally exposed by Western Horseradish Peroxidase (HRP) Substrate (WBKLS0500, Sigma).

**Immunohistochemistry and Immunofluorescence Analyses**

For immunohistochemistry, the samples were fixed in formalin, embedded in paraffin, and sectioned. The sections were de-paraffinised and blocked with 3% H2O2 (16B22C, Boster) for 20 min. To repair antigens, sections were boiled in sodium citrate buffer (C1032, Solarbio) for 20min and cooled at room temperature. After blocking in 5% bovine serum albumin (BSA) blocking solution (17E23C, Boster) for 30 min, the sections were incubated with primary antibodies overnight at 4 ℃ and secondary antibodies at 37 ℃ for 30min. Colour was developed using a diaminobenzidine (DAB) substrate kit (SK-4100, Vector). Sections were counterstained with haematoxylin solution (G1150, Solarbio) to stain nuclei. For immunofluorescence, the sections were de-paraffinised and boiled in citrate buffer before blocking with 3% BSA (A8010, Servicebio). The sections were then incubated with anti-STAP1 (1:200; HPA038529, Sigma 1:200) or anti-ARG1 (1:100; 93668, Cell Signaling Technology) overnight at 4 ℃ and then incubated with secondary antibodies at room temperature for 50 min. The above steps were repeated to stain CD11b (1:500, ab8878, Abcam) and CD45 (1:500; GB113885 for human, GB113886 for mouse, Solarbio). After incubation with 4,6-diamidino-2-phenylindole (DAPI) solution at room temperature for 10 min, the sections were sealed with an antifade mounting medium (S2100, Servicebio). For cell immunofluorescence, cells were cultured on glass slides. Then cells were stained with the following steps: fixed with 4% paraformaldehyde (BL539A, Biosharp) at 4 ℃ for 20 min, permeabilised with 0.5% Triton X-100 (9002-93-1, Sangon Biotech) at room temperature for 20 min and blocked with 1% BSA (A8010, Servicebio) at room temperature for 30 min. Sections were incubated with anti-ARG1 (1:200; 93668, Cell Signalling Technology) overnight at 4 ℃, fluorescent secondary antibody (SA00013, Proteintech) at 37 ℃ for 60 min, and DAPI solution (C0065, Servicebio) at room temperature for 10 min the next day. Finally, the sections were sealed with an antifade mounting medium (S2100, Servicebio). For immunohistochemistry, the histological score (H-score) was calculated as the proportion score × intensity score. For immunofluorescence, images were analysed using the HighPlex FL algorithm in HALO software (Indica Labs).

Chromatin immunoprecipitation (ChIP)

For the ChIP experiment, we used the SimpleChIP Enzymatic Chromatin IP Kit (9003, Cell Signaling Technology) according to the manufacturer’s instructions. Briefly, BV2 cells were added to 1% formaldehyde and incubated for protein-DNA complexes. After terminating the reaction, cells were washed and collected with PBS containing a protease inhibitor. Lysates were centrifugated at 2000 rpm for 5 min and sonicated to DNA fragments. The protein-DNA lysates were added with antibodies that against normal rabbit IgG, STAT3 (9139s, Cell Signaling Technology), and incubated with rotation overnight at 4 °C. Next, protein A agarose beads were added into the lysates and mixed for 2 h. Then, the complexes were reverse cross linked at 65 °C for 2 h in the presence of 0.2 M NaCl and Proteinase K. Subsequently, the precipitated DNA was purified using a centrifugal column. A total of 2 μl of the purified DNA was subjected to PCR amplification using primers that were derived from the Arg1 promoter. Primers were listed in Table. S3. The results were analyzed by expression as enrichment relative to input.
